# Issues with reporting and interpretation of Khan et al. 2021

**DOI:** 10.1186/s12879-022-07551-8

**Published:** 2022-06-22

**Authors:** Nicholas J. DeVito, Henry Drysdale

**Affiliations:** Nuffield Department of Primary Care Health Sciences, University of Oxford, Radcliffe Observatory Quarter, Woodstock Road, Oxford, OX2 6GG England

**Keywords:** Trials Transparency, Trial Reporting, Trial Registration, Convalescent Plasma, Covid-19

## Abstract

A recent publication in BMC Infectious Diseases concerning the use of convalescent plasma for the treatment of COVID-19 had a number of major issues. This correspondence details specific instances of unclear reporting as well as major omissions when discussing the context of the trial. These render the study's findings and conclusions misleading.

## Background

The published study from Khan and colleagues[1] on the treatment of COVID-19 patients in Pakistan with convalescent plasma (CP) contains a number of serious reporting issues. Unclear reporting and outcome definitions, failure to adequately discuss trial limitations, and the omission of large volumes of relevant literature from the discussion section render the study’s findings and conclusions misleading.

## Main text

The authors provide two trial registrations for this clinical trial: NCT04352751 on ClinicalTrials.gov; and IRCT20200414047072N1 on the Iranian Registry of Clinical Trials (IRCT). Trial registrations should be prospective to ensure no post hoc deviations can occur. The authors state that the first plasma infusions were given on 4 April 2020, however both trial registrations were submitted after the trial started: to ClinicalTrials.gov on 16 April 2020 and to IRCT on 28 April 2020. While BMC editorial policy does allow for the publication of retrospectively registered studies, it must be declared. The authors note the registration date was 28 April 2020 but do not make clear that this was retrospective [2].

Comparing the target sample size with the number of trial participants included raises additional concerns. The published report includes 50 COVID-19 patients, however the IRCT entry claims that this trial aimed for a target sample size of 357 and the ClinicalTrials.gov entry notes an estimated enrollment of 2000. The authors make no mention of the current recruitment status of the trial, whether further enrollment is planned, nor whether the trial was terminated early. Both registries offer conflicting information on the trial’s recruitment status: ClinicalTrials.gov says the trials is “Recruiting” but the IRCT states “Recruitment complete”. Neither registry entry has been updated since 2020. With no additional context or explanation offered, readers are left to wonder why the sample size is dramatically lower than planned, and if this compromises the interpretation of the results.

The outcomes reported in this study are inadequately defined, and different from those pre-specified on both ClinicalTrials.gov and IRCT. The published report states: "The primary and secondary endpoint of this study were, (a) improvement in clinical signs and symptoms and change in category of disease severity, (b) ability of CP to halt disease progression leading to invasive ventilation, respectively”, with no further details given in the report on the categories of disease severity nor the time-points at which outcomes were assessed. On ClinicalTrials.gov, the authors pre-specify one adequately defined primary outcome (“Change in COVID-19 severity status”) but do not pre-specify the secondary outcome that was reported. The IRCT contains five pre-specified primary outcomes: of these, four are inadequately pre-specified, with only vague detail given on the method of measurement for each outcome. While some of these overlap with the components of the primary outcome on ClinicalTrials.gov, it is not stated whether these pre-specified outcomes on the IRCT relate to that primary outcome, and the secondary outcome in the published article remains unspecified in the IRCT entry. The outcome reporting in this study therefore falls well short of the Consolidated Standards of Reporting Trials (CONSORT) reporting standards, which are endorsed by BMC Infectious Diseases, that state trial reports should include "Completely defined pre-specified primary and secondary outcome measures, including how and when they were assessed" and that authors should report “Any changes to trial outcomes after the trial commenced, with reasons.” [3, 4]

Lastly, the interpretation of this study is notably incomplete. The authors concede that the lack of a control group is a limitation in their study noting that “no inference on efficacy of CP can be drawn”, however they then conclude that plasma transfusion “seemed to be the safest modality of managing COVID-19 patients and the hall mark[sic] of this therapy is timely infusion and selection of category of recipients.” They cite only a single positive non-randomised study as context for their findings [5]. BMC Infectious Diseases accepted this study on 20 July 2021. The authors failed to consider either the Cochrane review (last updated May 2021) or a major review published in the *Journal of the American Medical Association* in February 2021, both of which examined randomized evidence and concluded, with confidence, that convalescent plasma “does not reduce mortality and has little to no impact on measures of clinical improvement” [6, 7]. In fact, it appears none of the 13 studies included in the Cochrane review, which included a total of 48,509 trial participants, are cited anywhere, including the landmark results from the RECOVERY trial [8]. All of these would have been available to the authors, editors and reviewers prior to acceptance. Readers are left with the impression that plasma is an effective therapy when substantial evidence to the contrary was left uncited [9].

## Conclusion

Overall, the reporting and interpretation of this study are extreme examples of deviations from best practice. If trial reports do not disclose and explain deviations from registrations, they can present readers with a misleading picture of the true results of the trial. Where trial outcomes are vaguely defined, the research community is unable to critically appraise or reproduce findings. Finally, the omission of large volumes of supporting evidence prevents readers from understanding the context of the reported trial, and allows for erroneous and misleading conclusions about a treatment’s efficacy and safety.

## Data Availability

Not applicable. This comment involved no original data or materials.

## Abbreviations

CP: Convalescent plasma
IRCT: Iranian Registry of Clinical Trials
CONSORT: Consolidated Standards of Reporting Trials
